# Bulk and nanoparticles of zinc oxide exerted their beneficial effects by conferring modifications in transcription factors, histone deacetylase, carbon and nitrogen assimilation, antioxidant biomarkers, and secondary metabolism in soybean

**DOI:** 10.1371/journal.pone.0256905

**Published:** 2021-09-08

**Authors:** Tahereh Mirakhorli, Zahra Oraghi Ardebili, Alireza Ladan-Moghadam, Elham Danaee

**Affiliations:** 1 Department of Biology, Garmsar Branch, Islamic Azad University, Garmsar, Iran; 2 Department of Horticulture, Garmsar Branch, Islamic Azad University, Garmsar, Iran; Bangabandhu Sheikh Mujibur Rahman Agricultural University, BANGLADESH

## Abstract

Nanoscience paves the way for producing highly potent fertilizers and pesticides to meet farmer’s expectations. This study investigated the physiological and molecular responses of soybean seedlings to the long-time application of zinc oxide nanoparticles (ZnO NPs) and their bulk type (BZnO) at 5 mg L^-1^ under the two application methods (I- foliar application; II- soil method). The ZnO NPs/BZnO treatments in a substance type- and method-dependent manner improved plant growth performance and yield. ZnO NPs transactionally upregulated the *EREB* gene. However, the expression of the *bHLH* gene displayed a contrary downward trend in response to the supplements. ZnO NPs moderately stimulated the transcription of *R2R3MYB*. The *HSF*-34 gene was also exhibited a similar upward trend in response to the nano-supplements. Moreover, the ZnONP treatments mediated significant upregulation in the *WRKY1* transcription factor. Furthermore, the *MAPK1* gene displayed a similar upregulation trend in response to the supplements. The foliar application of ZnONP slightly upregulated transcription of the *HDA3* gene, while this gene showed a contrary slight downregulation trend in response to the supplementation of nutrient solution. The upregulation in the *CAT* gene also resulted from the nano-supplements. The concentrations of photosynthetic pigments exhibited an increasing trend in the ZnONP-treated seedlings. The applied treatments contributed to the upregulation in the activity of nitrate reductase and the increase in the proline concentrations. ZnO NPs induced the activity of antioxidant enzymes, including peroxidase and catalase by averages of 48.3% and 41%, respectively. The utilization of ZnO NPs mediated stimulation in the activity of phenylalanine ammonia-lyase and increase in soluble phenols. The findings further underline this view that the long-time application of ZnO NPs at low concentrations is a safe low-risk approach to meet agricultural requirements.

## Introduction

Zinc (Zn) is an essential micronutrient for different kinds of living organisms, including plants. Zn performs significant fundamental roles in many biological processes such as protein synthesis, gene transcription, gene regulation, and metabolism of phytohormones [1–3]. In alkaline soils, plants face reduced access to Zn, a limiting factor that restricts crop yields and reduces the quality of crop-derived foods. Zn deficiency in crops not only restricts their productivity but also influences human health [4]. Hence, attempts have been made to introduce the novel, safe, and high-potent Zn fertilizers to counteract with Zn crisis and fulfill the agricultural requirements [3,4]. Taking sustainable agriculture into account, different modern technologies, in particular nanotechnology, offer novel strategies to meet the farmer’s expectations and the food demand of humans [3,4]. Nanotechnology paves the way for improving the efficiency of a vast array of commercial products such as fertilizers/pesticides in agriculture by exploitations of engineered nanoparticles (NPs), especially metal-based NPs. Nowadays, nano fertilizers can significantly contribute to fulfilling the target challenges in the sustainable agriculture and food industry. Numerous studies support this hypothesis that nanotechnology confers a considerable opportunity to meet agricultural requirements by providing novel highly-potent fertilizers/pesticides, reducing the excessive usages of conventional chemicals, and improving stress tolerance [2–7]. Contrary to this view, several reports underline the necessity of paying special attention to the cytotoxicity and ecotoxicity of nanoproducts [2,3,8–11]. Considering inadequate knowledge, the biological risk assessment of nano-based products should be, however, explored.

Taking zinc oxide nanoparticles (ZnO NPs; one of the most commonly used NPs) into account, the application of these nanomaterials improved growth and productivity in plant species, like *Datura stramonium* [2], wheat [12], and soybean [13]. Moreover, its application enhanced the production of secondary metabolites, like essential oils in Feverfew [14], phenolics in *Melissa officinalis* [15], alkaloids in *Datura stramonium* [2], and phenylpropanoids in tomato [4]. Exposure to ZnO NPs also affected phytohormones [3,16,17]. Besides, ZnO NPs mitigated the risk associated with diverse abiotic [7,18,19] and biotic [20] stress conditions. For instance, the foliar application of ZnO NPs mitigated the hazardous impact of chilling stress via affecting several stress-responsive genes in rice [7]. Therefore, the molecular assessment may fill the knowledge gap on mechanisms by which ZnO NPs may potentially confer stress tolerance in plants. On the other hand, ZnO NPs were associated with cytotoxicity and genotoxicity risk in Barley [11], *Arabidopsis* [9], and tomato [10,11].

Although a plethora of studies displayed the effects of ZnO NPs on vegetative growth at the early developmental phase, few researchers reported that these nanoparticles may positively or negatively affect plant productivity and yield at the reproductive stage, as well [4,13,21]. There, however, remains a scientific knowledge gap on how plant cells interact with ZnONP, thereby triggering these physiological responses. Several recent studies support this hypothesis that exposure of nanoparticles to plant cells may influence the transcriptome [2–5,7,11]. In this concern, investigation on plant transcriptional responses to ZnO NPs is necessary to elucidate the molecular mechanisms involved.

Transcription factors, mitogen-activated protein kinases (MAPKs), and epigenetic modification are critical mechanisms by which plant responses to both internal and external cues can be modulated [7,22–27]. Transcription factors are proteins that contribute to the regulation of a multitude of downstream genes by recognizing specific regulatory DNA sequences of domains during signal transduction. Among large families of transcription factors, some members, such as WRKY1 [7,26], R2R3MYB [4,7], bZIP [25], bHLH [4,22], and EREB [23] play fundamental roles in regulating growth, metabolism, and stress responses. In eukaryotic living organisms, including plant cells, MAPKs act as vital mediators contributed to the gene regulation events by controlling the phosphorylation of chromatin-related proteins, transcription factors, and co-regulatory components [3,27]. In addition to the transcription factors and MAPKs, the chromatin structure is another main factor in determining the gene accessibility to the transcriptional machinery. In this regard, histone deacetylases (HDA) are key enzymes that remove the acetyl group from histone proteins on DNA, thereby epigenetically determining gene accessibility and regulating gene expression [2]. However, there is inadequate information on whether nanoparticles can change the expression of transcription factors and histone deacetylases (*HDA*) genes [2,22,27]. Herein, the potential involvements of these highlighted genes in controlling plant responses to ZnO NPs and the bulk counterpart were explored.

Nearly a majority of existing scientific reports on ZnO NPs-mediated responses have monitored short-time responses of plants at the early developmental stage. However, little information is gained considering the effect of multiple applications of ZnO NPs at low concentrations, mainly to avoid the potential risk associated with the application of these nanomaterials. Taking plant responses to ZnO NPs into account, there is inadequate scientific knowledge in terms of [i] long-time application at low doses, [ii] molecular mechanism, especially at a signal transduction level, and [iii] comparative evidence on foliar and soil application methods. This study was carried out to respond following main research questions; i- does ZnONP trigger epigenetic responses? ii- Which of the target transcription factors are ZnONP responsive? iii- does the supplementation of nutrient solution with ZnONP induce a long-distance response in leaves? iv- Which application method is more efficient to improve plant growth, productivity, and immunity? v- is the plant’s response to nanoparticles different from the bulk counterpart?.

## Material and methods

### Treatments and experimental conditions

ZnO NPs (CAS Number: 1314-13-2; Stock number: US3590) were supplied by US research nanomaterials, Inc; 3302 Twig Leaf Lane Houston, TX 77084, USA). The nanoproduct had sizes ranging from 10 to 30 nm and displayed the acceptable zeta potential amount, (- 20 mV). Field emission scanning electron microscopy (FESEM) image, UV-Vis spectrum, and Zeta potential distribution graph are depicted in S1 Fig. Zinc oxide of Sigma company was utilized as a corresponding bulk control to provide comparative data.

Soybean (*Glycine max* L.) seed was provided by Seed and Plant Improvement Institute, Karaj, Iran. In this experiment, plants were cultured in a soilless medium with high leaching capacity to keep the concentration applied in the foliar method equal to the amount used in the soil method. Moreover, this experiment was performed in pots under a soilless medium consisting of Cocopeat: Perlite at a ratio of 60: 40 to avoid potential interference of soil factors. In this regard, the field capacity of the soilless medium was determined, which was 500 mL per pot. To keep the concentrations of the two methods equal, each pot was irrigated with a 300 mL solution containing BZnO/ZnO NPs per pot that was equal to the volume used for the spray method as well. To keep the concentrations almost constant and prevent over-accumulation of materials in the soil medium, the pots were irrigated with the nutrient solution above the field capacity (800 mL per pot) at intervals of the bulk or nano treatments for leaching the previous materials. In soil, especially heavy clay soil, the concentrations would exponentially increase and the concentration would not remain constant. Plants were cultivated under the same natural conditions in Garmsar (southeast of Tehran, Iran; relative humidity of 60%; day/night; light intensity: 90 μmol m^−2^ s^−1^; mean temperature: 27/16°C). Thirty-day-old plants were grouped in 5 treatment groups and treated with two concentrations of ZnONP or BZnO, including 0 and 5 mg L^-1^, 10 times with 72 h intervals under two different application methods; I- foliar application; II- soil application. it should be noted that the main experiment was designed according to the findings of the preliminary experiment. To fully disperse the nanoparticles, ZnO NPs dissolved in deionized water were ultra-sonicated using an ultrasonicator (40 kHz) for 30 min before the treatments. The treatment groups were called as follows; C: Control; BZnO-F: Foliar application of BZnO; ZnO NPs-F: Foliar application of ZnO NPs; BZnO-S: supplementation of nutrient solution with BZnO; ZnO NPs-S: supplementation of nutrient solution with ZnO NPs; The treated plants were harvested at two developmental stages; I- the first harvest was performed 48 h after the last treatments for assessment of biomass, molecular, and physiological characteristics; II- second harvest was carried out at the end of the plant life cycle to evaluate the crop yield.

### Real-time quantitative PCR (qRT-PCR)

Total RNA was purified from leaves by utilizing an RNA isolation kit (Denazist, Iran), Trizol reagent (GeneAll Biotechnology Co, South Korea), DEPC Water (BioBasic, Canada), and DNase I (Thermo Fisher, USA) according to the manufacturer’s protocol. Next, the RNA purity was confirmed based on the absorbance ratio at 260/280 nm (Nanodrop instrument; Thermo Scientific™NanoDrop Model 2000c). At the next step, the complementary DNA (cDNA) was prepared using a thermocycler instrument (PEQLAB, 96Grad) was utilized to perform. Moreover, Oligo7 and AllelID software were utilized to design the forward and reverse primer sequences for each target gene investigated. The forward and reverse sequences of primers for the monitored genes, including histone deacetylase (*HDA*, XM_006592247.2), heat stress transcription factor (Hsf-34; XM_003550218.2), ethylene-responsive element-binding protein (*EREB*; NM_001349033.1), *MYB* transcription factors (*R2R3MYB*; NM_001370267.1), *WRKY1* (EU019552.1), basic/helix-loop-helix (bHLH; KT031116.1), Catalase (*CAT*; NM_001247898), mitogen-activated protein kinase 1 (*MAPK1*; AF104247), and Elongation factor (a housekeeping gene) are presented in Table 1. To investigate transcription variation among the treatment groups, the real-time quantitative PCR (Applied Biosystems StepOne™ Real-Time PCR) was conducted under a cycling program of 94°C: 120 s, 94°C: 15s, 57°C: 25 s, and 72°C: 20 s. The relative transcription of the target genes was estimated using the equation of 2^-ΔΔCT^ in which ΔCT refers to subtracting the internal control CT amount from the CT value of each gene investigated [4,22,28].

**Table 1:** The forward and reverse sequences of primers for target genes and Elongation factor (a housekeeping gene).

Primer name	Sequence (5’-3’)	Tm (°C)	Length (nt)	Product (bp)
*Ef1a*-F	TGAGAAGGAAGCTGCTGAGA	59	20	157
*Ef1a*-R	TCCAGGCGCATCAATAACTG	59	20	
*CAT*-F	GTCAGAAAGCCATGGATCCC	59	20	139
*CAT*-R	TCCAGCAGAATTGGACCTCT	59	20	
*bHLH*-F	AATCTCCCCATGTCGGCTAT	59	20	160
*bHLH*-R	TCTTGCCTCCTTTGCCTTTC	59	20	
*Hsf34*-F	GCCTGCATCGATTTTGGTTG	59	20	130
*Hsf34*-R	CAAATCCTCCATCGGCACAT	59	20	
*R2R3MYB*-F	AAAGCAAGCTAGGGCCAAAA	59	20	170
*R2R3MYB*-R	ACCAATTCCCAGACCCTGAT	59	20	
*EREB*-F	CTCTCGTGGATCAGCAACTG	59	20	134
*EREB*-R	TTCTTGGGTCACGGATCTCA	59	20	
*WRKY1*-F	CAGAAGCAGAAGCAGTGTCA	59	20	118
*WRKY1*-R	GAAATGGTGCAACTTGAGCG	59	20	
*MAPK1*-F	GTACCAGATTCTTCGTGGGC	59	20	192
*MAPK1*-R	AATTCGGGAGCTCTGTACCA	59	20	
*HDA*-F	CCTGGTGCAATAGTTCTGCA	59	20	135
*HDA*-R	AGTGACCAGCAATGGCAAAT	59	20	

### Enzyme extraction and assessments of activities of enzymes

The possible differences in some important enzymes involved in the antioxidant system, nitrogen assimilation, and phenylpropanoid metabolism were investigated. These enzymes were catalase and peroxidase, nitrate reductase, and phenylalanine ammonia-lyase (PAL). First, the leaves were homogenized in the phosphate buffer (0.1 M; pH 7.5) supplemented with ascorbate and Na_2_EDTA. The next step was the centrifugation of the resulting homogenates at 4°C. After that, the resulting supernatants were kept at − 80°C for the enzymatic assessments.

The catalase activity in leaves was spectrophotometrically investigated by recording the decrease in absorbance at 240 nm per min (ΔA 240 nm) in an enzyme reaction medium containing phosphate buffer and H_2_O_2_. Then, the micromole of the degraded substrate (H_2_O_2_) was calculated using extinction coefficient (ε = 39.4 mM^-1^ cm^-1^). Definition of a unit of catalase activity is the amount of enzyme that is required to decompose 1.0 μmole of the substrate (H_2_O_2_) per min. To estimate peroxidase activity, the amount of increase in the absorbance at 470 nm (ΔA 470 nm) per min following the addition of enzyme extract into the reaction medium containing guaiacol, phosphate buffer, and H_2_O_2_. Enzyme activity was calculated using the extinction coefficient (ε = 26.6 mM^-1^ cm^-1^). Unit enzyme activity is expressed as the amount of enzyme that is required for the oxidation of guaiacol into the tetra-guaiacol at 470 nm. To evaluate the nitrate reductase activity, the common protocol provided by Sym [29] was conducted. After keeping the reaction mixture (potassium nitrate, enzyme extract, and phosphate buffer) at dark conditions for 1 h, Griess reagent I and Griess reagent II were added. The procedure was continued by recording the absorbance at 540 nm. The nitrite concentration was quantified based on the standard curve of sodium nitrite and the enzyme activity was expressed in terms of μmol NO_2_^−^ h^-1^ g^−1^ fw. PAL activity was estimated based on the conversion rate of phenylalanine to cinnamate during 1 h after the addition of enzyme extract to the reaction medium [30]. The reaction mixture containing phenylalanine and Tris-HCl buffer was kept at 37 ˚C for 1 hour. After that, the conversion reaction of phenylalanine to cinnamate was stopped by adding HCl. Finally, the cinnamate concentration PAL activity was quantified based on the standard curve equation of cinnamate. The PAL activity was finally expressed in terms of microgram cinnamate per hour per gram fresh weight (μg Cin. h^−1^ g^−1^ fw).

### Quantification of photosynthetic pigments, proline, and soluble phenols

The ZnONP/BZnO-associated variations in the concentrations of photosynthetic pigments, including chlorophyll a (Chl a), chlorophyll b (Chl b), and carotenoids were investigated. The photosynthetic pigments were purified by homogenizing the leaves in the acetone solvent inside the mortar. The absorptions of filtrated acetonic extracts were spectrophotometrically recorded at wavelengths, including 470, 663, and 645 nm. The concentrations of Chl a, Chl b, and carotenoids were calculated using the equations presented by Lichtenthaler and Welburn [31].

To determine the concentration of proline, the sulfa salicylic acid of 3% (w/v) was utilized to extract proline. The reaction was initiated by adding 2 mL leaf extract to the reaction mixture containing 2 mL ninhydrin reagent and an equal volume of glacial acetic acid. Then, the reaction continued by heating under the water bath for 1 h and followed by immediately cooling down at an ice bath. The next step was the addition of toluene solvent and vigorously shaking by a vortex instrument. Finally, the optical density of the resulting toluene phase was spectrophotometrically monitored at 520 nm and the concentration was quantified based on the standard curve of proline [32].

To measure total soluble phenols, leaf extract was prepared with homogenizing leaves in ethanol solvent (80%) and incubating in a boiling water bath. Then, leaf extract was added to a mixture containing the Folin-Ciocalteu reagent and saturated Sodium carbonate (21%). The reaction was associated with developing a blue color. After centrifugation, the optical density of the supernatant was recorded at the wavelength of 760 nm [32]. The standard curve of tannic acid was served to determine the concentration of soluble phenols.

### Statistical analysis

The experimental design was completely randomized. All data were subjected to analysis of variance (ANOVA) using GraphPad software. The differences among the mean values of treatment groups were statistically compared according to the Tukey test analysis at a level of 5% of probability. The heatmap correlation matrix was also prepared using GraphPad software to express the potential correlation between the traits monitored.

## Results

**Fig 1:**
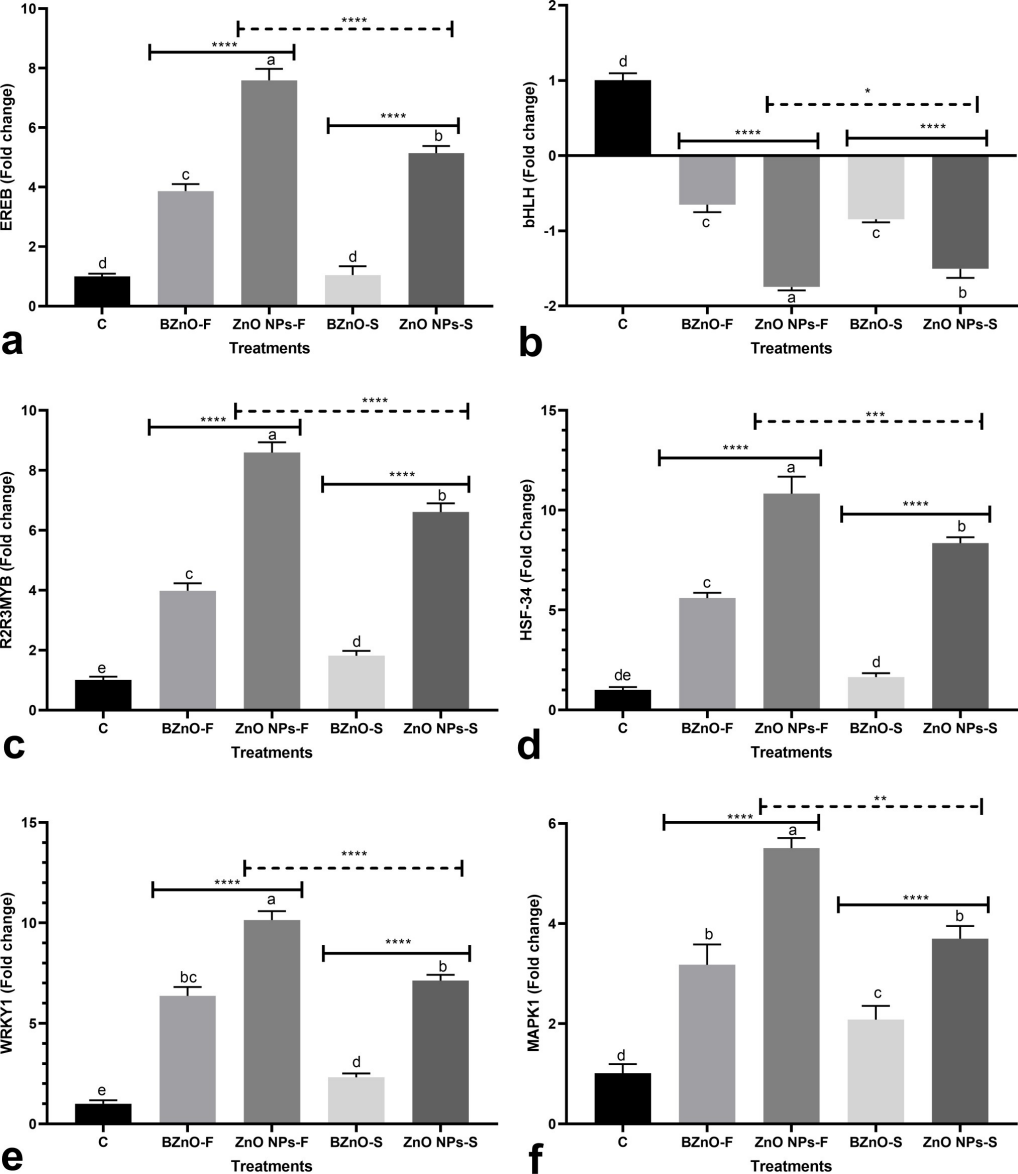
The effects of BZnO or ZnONP in two application methods (foliar and supplementation of nutrient solution) on the transcription of several genes, including *EREB* [a], *bHLH* [b], *R2R3MYB* [c], *HSF*-34 [d], *WRKY1* [e], and *MAPK1* [f]; The treatment groups: C: Control; BZnO-F: Foliar application of BZnO; ZnO NPs-F: Foliar application of ZnO NPs; BZnO-S: Supplementation of nutrient solution with BZnO; ZnO NPs-S: Supplementation of nutrient solution with ZnO NPs; Different letters on the columns refer to statistically significant differences according to Tukey’s multiple comparisons test. A comparison between the two BZnO and ZnO NPs groups at the same method is presented by placing an asterisk [*] on the line, while asterisks [*] on the dashed lines define the comparison between two ZnO NPs-F and ZnO NPs-S groups. ns: Non-significant; *: 0.01 < p ≤ 0.05; **: 0.001 < p ≤ 0.01; ***: 0.0001 < p ≤ 0.001; ****: p ≤ 0.0001.

The BZnO-F, ZnO NPs-F, BZnO-S, and ZnO NPs-S treatments significantly increased biomass accumulation by 24.8%, 52.7%, 14%, and 37.2%, respectively, in the shoot (Table 2). The root biomass displayed a similar upward trend in response to the BZnO-F (16.4%), ZnO NPs-F (34.9%), BZnO-S (31.7%), and ZnO NPs-S (48%) treatments (Table 2). The applied treatments significantly enhanced leaf fresh mass by an average of 37.7% over the control (Table 2). With a similar trend, both supplementation of nutrient solution and foliar application, especially the latter method, were slightly increased the number of leaves (Fig 1D). The BZnO-F (27%), ZnO NPs-F (60%), BZnO-S (15%), and ZnO NPs-S (43%) treatments contributed to improvement in crop yield, indicated by the significantly higher numbers of pods compared to the control (Table 2). The foliar application of the supplements slightly decreased the pod biomass which was the non-significant difference (Table 2). The BZnO-F and ZnO NPs-F treatments significantly increased Zn concentrations in leaves by 27% and 34%, respectively over the control group (Table 2). While the BZnO-S and ZnO NPs-S treatments led to a significant increase in leaf Zn contents by 11.5% and 19.3% compared with the control (Table 2). Likewise, the BZnO-F (23%), ZnO NPs-F (30%), BZnO-S (9.7%), and ZnO NPs-S (15%) treatment groups had higher concentrations of Zn in seeds in comparison with the control (Table 2). The foliar application method was a more efficient way to improve Zn bioaccumulation than the soil method (Table 2).

**Table 2:** The effect of BZnO or ZnO NPs and application methods on various characteristics related to growth, yield, and Zn concentration.

Treatments	Shoot Fresh Mass (g)	Root dry mass (g)	Leaf Fresh mass (g)	Number of leaves	Numbers of Pods	Pod weight (g)	Leaf Zn (μgg^-1^dw)	Seed Zn (μgg^-1^dw)
C	43 ± 2^e^*	1.9 ± 0.13^d^	4.5 ± 0.31^e^	16.3 ± 0.57^c^	30.6 ± 1.5^e^	1.7 ± 0.035^ab^	204 ± 9.7^e^	135.8 ± 4.3^e^
BZnO-F	54.7 ± 1.5^bc^	2.2 ± 0.04^c^	6.47 ± 0.2^b^	20.6 ± 1.15^b^	39 ± 1^c^	1.55 ± 0.08^bc^	260 ± 3^b^	168 ± 2.96^b^
ZnO NPs-F	65.6 ± 2.2^a^	2.6 ± 0.05^b^	7.2 ± 0.29^a^	24.3 ± 0.61^a^	51.3 ± 2.1^a^	1.53 ± 0.11^bc^	276 ± 4.5^a^	177.7 ± 2.7^a^
BZnO-S	48.9 ± 1.7^d^	2.5 ± 0.06^b^	5.38 ± 0.11^cd^	19.34 ± 1.2^b^	34.7 ± 0.6^d^	1.75 ± 0.07^a^	228 ± 2.2^d^	149 ± 3.6^d^
ZnO NPs-S	2.82 ± 2.3^b^	2.8 ± 0.08^a^	5.9 ± 0.15^bc^	24.7 ± 1.3^a^	44 ± 1.3^b^	1.66 ± 0.06^ab^	244 ± 3.6^c^	157.3 ± 2.5^cd^

*: mean ± standard deviation (SD); Mean values followed by a different letter (s) are significantly different according to Tukey’s test.

The BZnO-F, ZnO NPs-F, and ZnO NPs-S treatments significantly upregulated *EREB* by 3.8, 7.6, and 5.14-fold, respectively. While the difference between the BZnO-S and control groups was not statistically significant (Fig 1A). The expression of *bHLH* was slightly downregulated in response to the applied supplements (Fig 1B). The BZnO-F and BZnO-S treatments led to a slight significant up-regulation in the expression of *R2R3MYB* by an average of 2.9-fold (Fig 1C). However, the ZnO NPs-F and ZnO NPs-S treatments moderately stimulated the transcription of *R2R3MYB* by an average of 7.6-fold relative to the control (Fig 1C). The *HSF-34* gene was significantly upregulated in response to the BZnO-F, ZnO NPs-F, and ZnO NPs-S treatments by an average of 8.3-fold, while the BZnO-S treatment made no significant change in comparison to the control (Fig 1D). The BZnO-F, ZnO NPs-F, BZnO-S, and ZnO NPs-S treatments mediated significant upregulation in *WRKY1* by 6.3, 10.1, 2.3, and 7.1-fold, respectively compared to the control (Fig 1E). *MAPK1* also displayed a similar upregulation trend in response to the supplements (Fig 1F).

**Fig 2:**
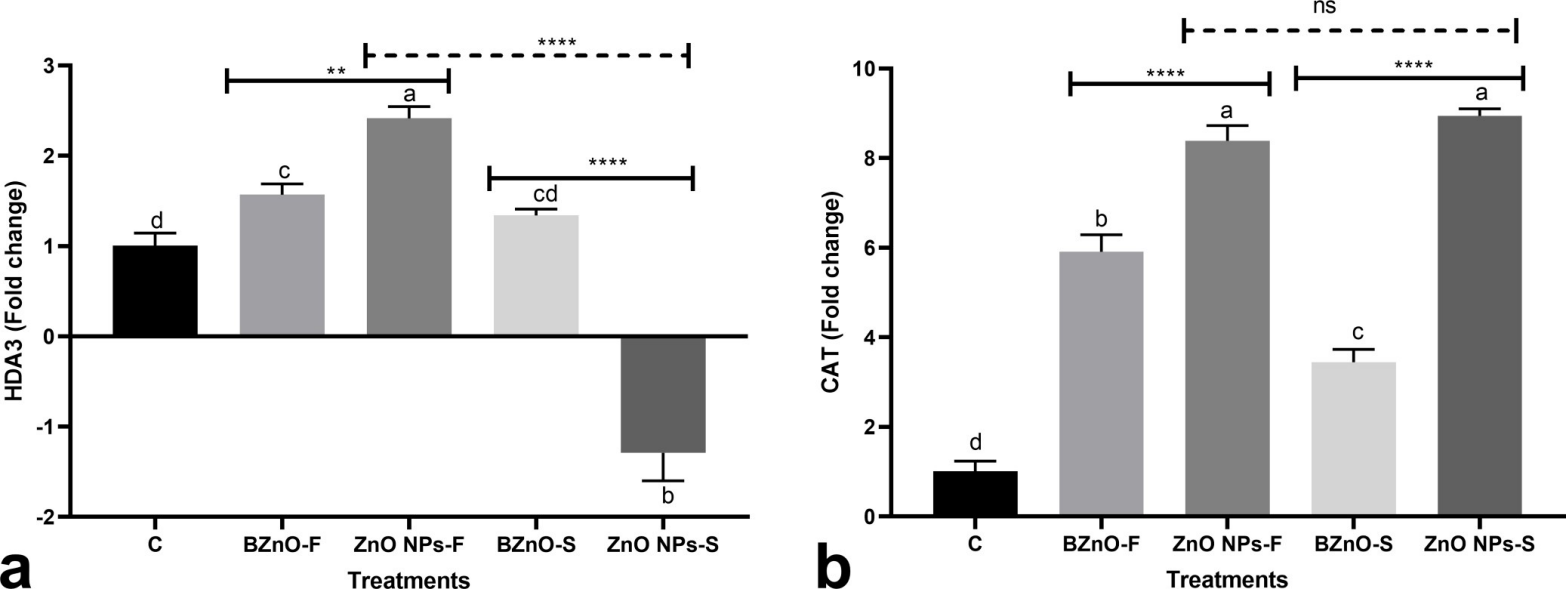
The transcriptional responses of *HDA3* [a] and *CAT* [b] genes following the BZnO or ZnONP treatments. The treatment groups: C: Control; BZnO-F: Foliar application of BZnO; ZnO NPs-F: Foliar application of ZnO NPs; BZnO-S: Supplementation of nutrient solution with BZnO; ZnO NPs-S: Supplementation of nutrient solution with ZnO NPs; Different letters on the columns refer to statistically significant differences according to Tukey’s multiple comparisons test. A comparison between the two BZnO and ZnO NPs groups at the same method is presented by placing an asterisk [*] on the line, while asterisks [*] on the dashed lines define the comparison between two ZnO NPs-F and ZnO NPs-S groups. ns: Non-significant; *: 0.01 < p ≤ 0.05; **: 0.001 < p ≤ 0.01; ***: 0.0001 < p ≤ 0.001; ****: p ≤ 0.0001.

The BZnO-F and ZnO NPs-F slightly upregulated *HDA3* by an average of 1.9-fold, while this gene showed a contrary slight downregulation trend in response to the ZnO NPs-S treatment in comparison to the control (Fig 2A). The BZnO-F, ZnO NPs-F, BZnO-S, and ZnO NPs-S treatments significantly upregulated the CAT gene by 5.9, 8.4, 3.4, and 8.9-fold, respectively relative to the control (Fig 2B).

**Fig 3:**
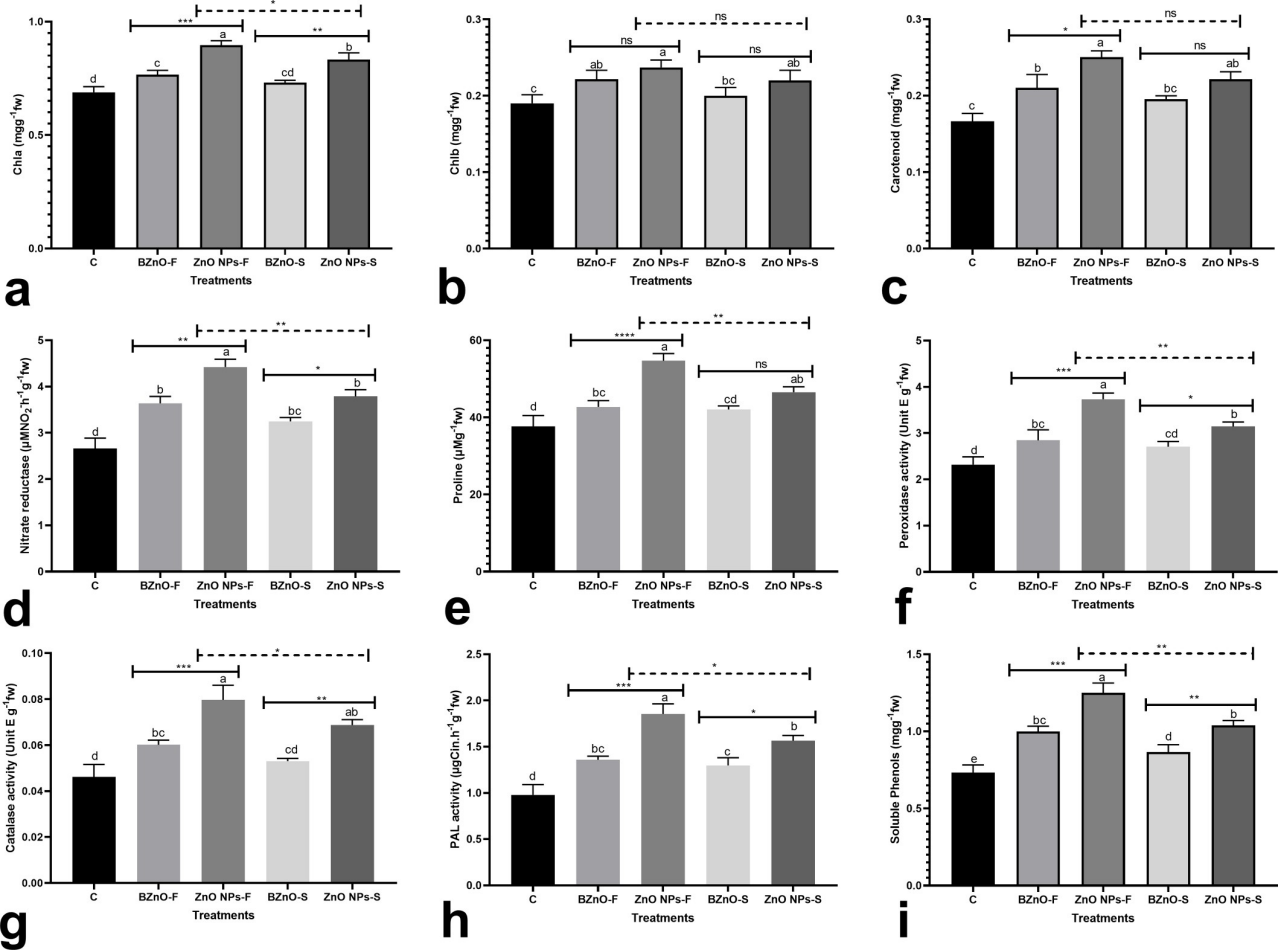
The BZnO or ZnONP-mediated changes in several physiological traits, including Chl a [a], Chl b [b], carotenoids [c], nitrate reductase [d], proline [e], peroxidase activity [f], catalase activity [g], PAL activity [h], and soluble phenols [i]; The treatment groups: C: Control; BZnO-F: Foliar application of BZnO; ZnO NPs-F: Foliar application of ZnO NPs; BZnO-S: Supplementation of nutrient solution with BZnO; ZnO NPs-S: Supplementation of nutrient solution with ZnO NPs; Different letters on the columns refer to statistically significant differences according to Tukey’s multiple comparisons test. A comparison between the two BZnO and ZnO NPs groups at the same method is presented by placing an asterisk [*] on the line, while asterisks [*] on the dashed lines define the comparison between two ZnO NPs-F and ZnO NPs-S groups. ns: Non-significant; *: 0.01 < p ≤ 0.05; **: 0.001 < p ≤ 0.01; ***: 0.0001 < p ≤ 0.001; ****: p ≤ 0.0001.

**Fig 4:**
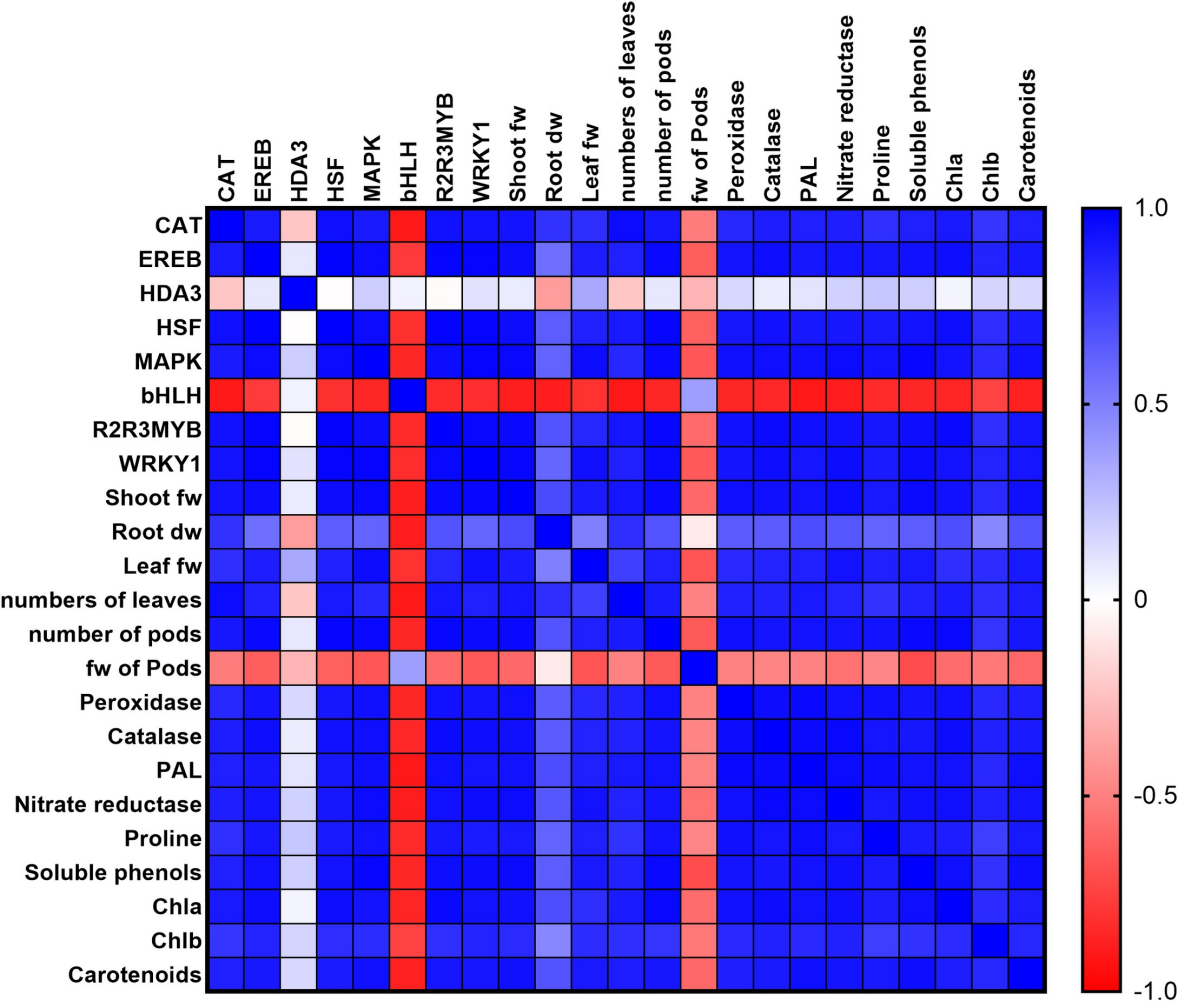
Heatmap correlation matrix among molecular and physiological traits investigated.

The concentrations of photosynthetic pigments, including Chl a, Chl b, and carotenoids exhibited an upward trend in response to the supplements (Fig 3A–3C). Among the treatment groups, the ZnO NPs-F group contained the highest concentration of photosynthetic pigments. The BZnO-F, ZnO NPs-F, BZnO-S, and ZnO NPs-S treatments contributed to the significant upregulation in the activity of nitrate reductase by an average of 36.8% compared to the control (Fig 3D). The BZnO-F, ZnO NPs-F, and ZnO NPs-S treatments contributed to the significant increase in the proline concentrations by 13.4%, 45.1%, and 23.6%, respectively, compared with the control (Fig 3E). The BZnO-F, ZnO NPs-F, BZnO-S, and ZnO NPs-S treatments induced the activity of the peroxidase enzyme by 22.7%, 61%, 16.8%, 35.6%, respectively, over the control (Fig 3F). Likewise, the activity of the catalase enzyme was upregulated by an average of 41% in comparison with the control (Fig 3G). The BZnO-treated plants showed higher activities of PAL enzyme by an average of 35.8%, while the ZnONP-supplemented seedlings led to up-regulation in the activity of this enzyme by 74% over the control (Fig 3H). The soluble phenols displayed a similar upward trend by an average of 41.7% (Fig 3I). The heatmap correlation matrix presented in Fig 4, clearly exhibits the strong significant correlation among the majority of molecular and physiological characteristics investigated in this study.

## Discussion

The findings confirmed that the supplements in substance type- and application method-dependent manners influenced soybean seedlings at different growth, physiological, and molecular aspects. Scholars firmly believe that Zn plays fundamental regulatory roles in a multitude of biological processes such as chromatin architecture, meristem performance, cell division, cell cycle, and metabolism of phytohormones [3]. In both application methods, the ZnO NPs or their bulk form not only did not show phytotoxic impacts but also improved plant growth performance and metabolism. The results confirmed that both material type and application method are two vital factors affecting plant responses and the efficacy of nanomaterials. The foliar application method was more efficient than the soil method, indicated by the observed significant differences in various physiological and molecular traits investigated. These findings further underline the opinion that the multiple foliar applications of ZnO NPs at low concentrations are safe low-risk approaches to improve growth, physiology, immunity, and productivity in crops. Moreover, it can be concluded that the potential risk of the foliar method is lower than the soil method as nanoparticles may adversely affect the soil microbiome [3]. The recorded physiological responses are consistent with the findings of Vafaee et al., [2] in *Datura stramonium* and Pejam et al., [4] in tomato. Pejam et al., [4] recently reported that the foliar utilization of ZnO NPs enhanced biomass, yield, nutritional status, and metabolism in the tomato plant.

Taking carbon metabolism into account, the supplements, especially the nano form, enhanced photosynthetic pigments, among which the increase in carotenoids is a vital protective mechanism, thereby improving plant tolerance to photoinhibition phenomenon during the stress conditions. Considering the observed variation in nitrate reductase activity and proline content, it was concluded that the efficacy of the nanoform in both application methods was more than the bulk to enhance nitrogen metabolism. The statistical analysis also confirmed the strong correlation among these markers in carbon and nitrogen primary metabolism. ZnO NPs at non-toxic doses were associated with improvement in nutritional status [4,15], carbon assimilation through photosynthesis [4,33], and nitrogen metabolism [2,4,33]. Contrary to these reports, the phytotoxicity of ZnO NPs contributed to the down-regulation in the transcription of several genes involved in the chlorophyll synthesis and photosystem structure, whereas ZnO NPs affected the production of carotenoids by upregulating genes such as *PSY* in *Arabidopsis* [9].

One of the proposed functions for ZnO NPs is to improve the resistance of plants to stress as one of the main needs of agriculture. Several studies exhibited the effectiveness of using ZnO NPs in activating defense system, stimulating secondary metabolism, and mitigating the risk of various stress conditions, such as cadmium in wheat [34], salinity in tomato [18], drought in sorghum [35], and chilling stress in rice [7]. Moreover, ZnO NPs treatment conferred systemic resistance against Tobacco Mosaic Virus through upregulation in the transcription of PR-1 (salicylic acid marker gene), *CHS*, *POD*, and *PAL* genes, implying activation of defense machinery [20]. However, the underlying molecular mechanisms by which ZnO NPs may confer these responses remain unknown. For this reason, this study intended to address the ZnO NPs-mediated changes in antioxidant biomarkers, secondary metabolism (PAL and soluble phenols), and molecular indices involved in regulating plant stress responses and developmental programs.

The nano form, especially in the foliar method, was more capable of remodeling the transcription of the target genes than the bulk. However, the soil application method also displayed high efficiency to trigger long-distance (root to shoot) signaling, thereby modifying the expression of genes investigated. Although the Zn concentration in the leaves of the BZnO-F group was higher than the ZnO NPs-S group, the effectiveness of the ZnO NPs-S treatment in affecting the expression of the target genes was higher than the BZnO-F group. This finding indirectly suggests the opinion that the plant response to ZnO NPs may, at least in part, resulted from the exclusive signaling of the nanomaterials rather than the effects of Zn ions. The foliar application of ZnO NPs upregulated the *HDA* gene, while the soil application method reversed the expression of this gene, implying the critical roles of the application methods. Little is known about epigenetic responses to nanoparticles. Several epigenetic responses such as DNA methylation [25,26,27] and histone deacetylase [2,4,27] have been reported following the application of nanomaterials. It is worth mentioning that this is the first investigation highlighting how the different application methods may be associated with differential epigenetic responses.

This study, therefore, provides a piece of molecular evidence on how exposure to ZnO NPs may contribute to activation of the defense system and stress tolerance via mediating changes in the expression of transcription factors, MAPKs, and epigenetic modification. It appears that transient alteration in cellular redox status, changes in phytohormones, and redox-based regulation are potentially responsible for the observed alterations in the expression of the investigated genes and activation of the plant immunity system. There is no denying the fact that MAPKs along with transcription factors are major components of regulatory networks by which a wide spectrum of downstream genes are regulated. It has been well documented that HSFs [4,22,36], EREB [23], WRKY1 [7,26,37], and R2R23MYB [2,4,7,22] are largely involved in the regulation of genes of the defense system and metabolism. The ZnO NPs-mediated transcriptional responses have been supported by several molecular evidence in different plant species such as *Datura stramonium* [2], maize [38], brassica [5], tomato [4,33], and rice [7]. The molecular assessment revealed that several transcription factors, including *OsbZIP52*, *OsMYB4*, *OsMYB30*, *OsNAC5*, *OsWRKY76*, and *OsWRKY94*, were upregulated in response to the foliar application of ZnO NPs, thereby improving plant resistance against chilling stress in rice [7]. ZnO NPs also induced genes that contributed to the antioxidant system, such as *OsCATA*, *OsCATB*, *OsPRX65*, *OsPRX89*, *OsPRX11*, *OsCu*/*ZnSOD1*, *OsCu*/*ZnSOD2*, and *OsCu*/*ZnSOD3* [7]. The involvement of microRNAs in plant responses to nanoparticles has been supported in barley [11] and tomato [24]. It has been well confirmed that microRNAs have close crosslinks with transcription factors and chromatin remodeling systems [27].

In brassica, ZnO NPs in a dose-dependent manner influenced the transcriptions of the cellular expression of cation efflux transporter gene (*BjCET2*) and metal tolerant protein (*BjMTP*) and [5], implying the transcriptional involvement of ZnO NPs in plant nutrition. The application of ZnO NPs was associated with slight changes in expression of the transcription factors (by approximately 5-fold) and activation of plant immunity. These responses might result from a transient change in redox status and subsequent redox-based control of genes. The transcriptions of stress-responsive genes are modulated by redox-based management, in particular at transcriptional and post-translational levels [27]. Besides, these molecular responses explain how the application of ZnO NPs at optimum doses potentially mitigates the risk of stress conditions. The transactional pattern of bHLH displayed a contrary trend compared to the other genes investigated. This response can be a significant feedback regulatory marker, thereby controlling the expression of downstream genes. Current evidence supports the involvement of bHLHs in a multitude of biological events, including phytohormone signaling, stomatal conductance, light signaling, shoot organogenesis and morphogenesis, root development, and stress/stimulus responses [34,39]. Nakata et al., [40] provided molecular evidence on how bHLH acts as a transcriptional repressor of jasmonate and adversely influenced signaling of jasmonate, a phytohormone involved in the regulation of the tradeoff between plant growth and stress responses. In tobacco, ZnO NPs contributed to the morphological, physiological, and anatomical responses by adjusting auxin levels, tissue differentiation, metabolism, and immune system [41]. As is well known, phytohormones have close dual crosslinks with MAPKs and transcription factors. It may be, therefore, expected that the ZnO NPs-mediated changes in expression of the target genes correlate with alteration in phytohormones and signaling molecules such as nitric oxide, H_2_O_2_, and H_2_S; needs to be explored in the future.

Overall, the physiological and molecular assessments provide convincing evidence comparing the efficacy of ZnO NPs and bulk ZnO to modify growth performance, yield, photosynthesis, nitrogen assimilation, secondary metabolism markers, antioxidant biomarkers, and transcriptional responses. This study also provided a piece of molecular evidence indicating the fundamental role of the type of substance and the application method in influencing epigenetic modification.

## Conclusion

Nanoscience paves the way for producing highly potent fertilizers and pesticides to meet farmer’s expectations. This study was conducted to fill the knowledge gap through monitoring the physiological and molecular responses of soybean seedlings to the long-time application of ZnO NPs and their bulk type at low doses under the two-application method. In both application methods, the ZnO NPs or their bulk form (especially the nano type) not only did not show phytotoxic impacts but also improved plant growth performance and metabolism. The findings further underline the opinion that the multiple application of ZnO NPs at low concentrations is a safe low-risk approach to meet agricultural requirements. The molecular findings manifest the hypothesis that exposure to ZnO NPs may widely affect the expression of transcription factors and subsequently their downstream genes, thereby influencing wide aspects of plant growth, metabolism, productivity, and immunity. The nano-form, especially in the foliar method, was more capable of remodeling the transcription of the target genes than the bulk. However, the supplementation of nutrient solution also displayed high efficiency to trigger long-distance signaling, thereby modifying the expression of genes investigated. This study is the first investigation highlighting how the different application methods may be associated with differential epigenetic responses. Taken collectively, ZnO NPs at low doses can be considered as a highly potent substance for potential utilization in plant-related sciences and industries.

## Supporting information

S1 Fig The physiocochemical traits of ZnO NPs, including FESEM image (a), UV-Vis spectrum (b), and Zeta potential distribution graph (c).
(TIF)
