# Supplementary figures and images for: Bulk and nanoparticles of zinc oxide exerted their beneficial effects by conferring modifications in transcription factors, histone deacetylase, carbon and nitrogen assimilation, antioxidant biomarkers, and secondary metabolism in soybean
Source: PLoS One. 2021 Sep 8;16(9):e0256905. doi: 10.1371/journal.pone.0256905 (PMC8425562; doi:10.1371/journal.pone.0256905)

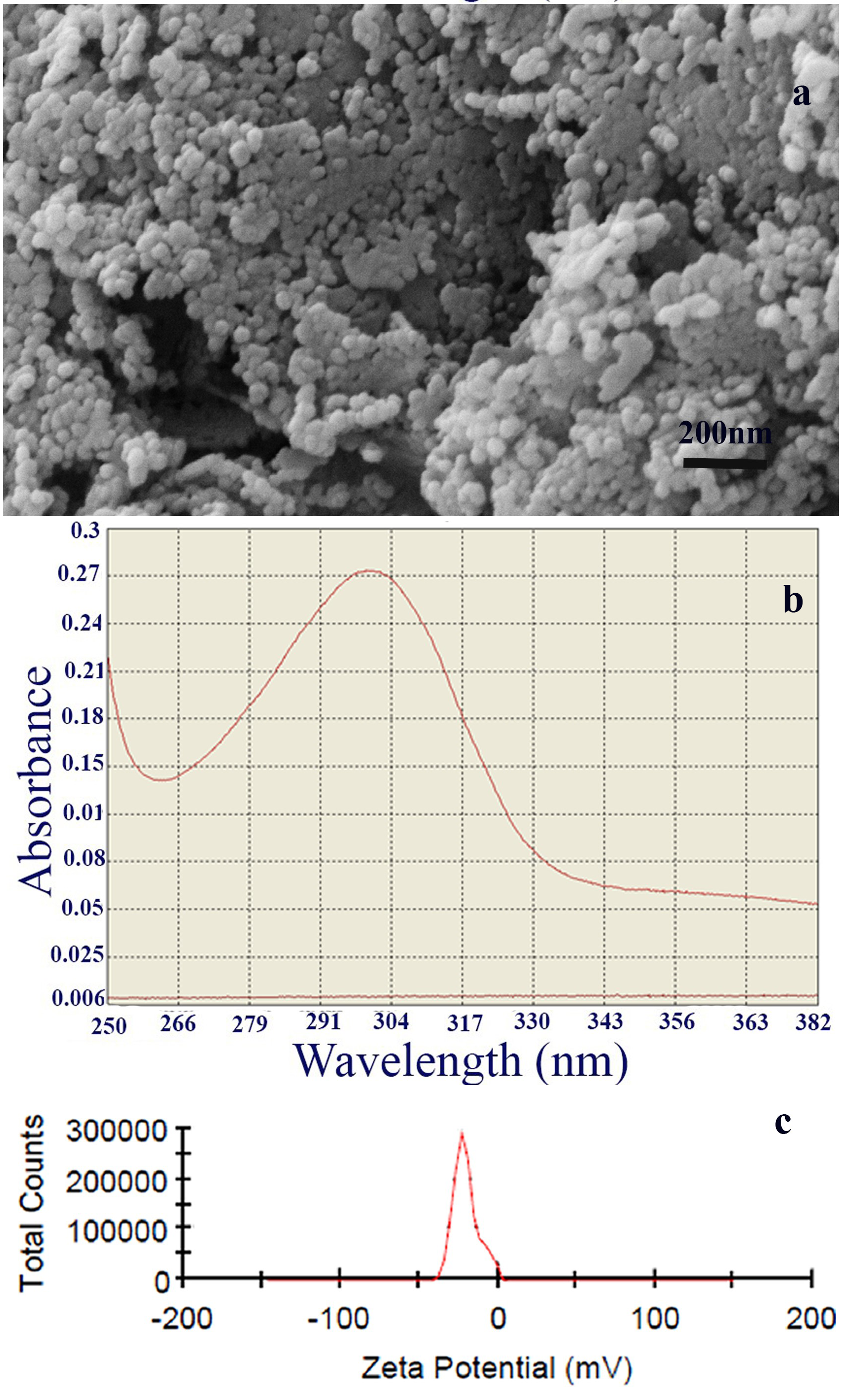

Supplement: S1 Fig — The physiocochemical traits of ZnO NPs, including FESEM image (a), UV-Vis spectrum (b), and Zeta potential distribution graph (c). (TIF) [file pone.0256905.s001.tif]
